# Biobased epoxy reactive diluents prepared from monophenol derivatives: effect on viscosity and glass transition temperature of epoxy resins†

**DOI:** 10.1039/d3ra01039b

**Published:** 2023-05-16

**Authors:** Samuel Malburet, Hugo Bertrand, Cécile Richard, Colette Lacabanne, Eric Dantras, Alain Graillot

**Affiliations:** a SPECIFIC POLYMERS 150 Avenue des Cocardières 34160 Castries France samuel.malburet@specificpolymers.fr; b CIRIMAT, Université Toulouse III – Paul Sabatier 118 Route de Narbonne 31062 Toulouse Cedex 09 France

## Abstract

The use of reactive diluents is undeniably of paramount importance to develop epoxy resins which would meet more demanding and restrictive processes and applications in terms of viscosity and glass transition temperature. In the context of developing resins with low carbon impacts, 3 natural phenols namely carvacrol, guaiacol and thymol were selected and converted into monofunctional epoxies using a general glycidylation procedure. Without advanced purification, the developed liquid-state epoxies showed very low viscosities of 16 cPs to 55 cPs at 20 °C, which could be further reduced to 12 cPs at 20 °C when purification by distillation is applied. The dilution effect of each reactive diluent on DGEBA's viscosity was also assessed for concentrations ranging from 5 to 20 wt% and compared to commercial and formulated DGEBA-based resin analogues. Interestingly, the use of these diluents reduced the initial viscosity of DGEBA by a factor of ten while maintaining glass transition temperatures above 90 °C. This article provides compelling evidence of the possibility of developing new sustainable epoxy resins with characteristics and properties that can be fine-tuned by only adjusting the reactive diluent concentration.

## Abstract 2

The use of reactive diluents is undeniably of paramount importance to develop epoxy resins which would meet more demanding and restrictive processes and applications in terms of viscosity and glass transition temperature.

## Graphical abstract

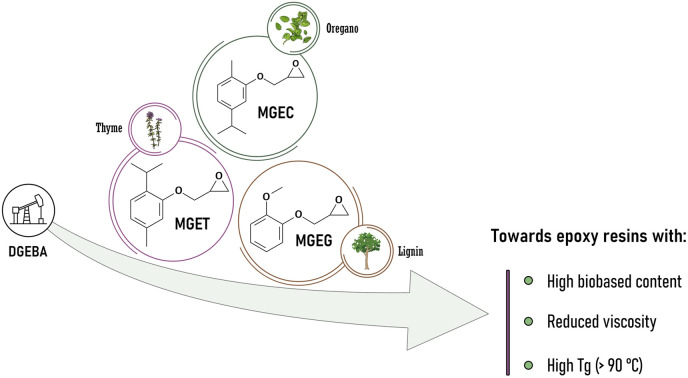

## Introduction

The vast majority of epoxy resins available on the market are derived from the diglycidyl ether of bisphenol-A also well-known as DGEBA. This market dominance is a result of a combination of its low cost and adequate-to-superior performance in many applications due to its high chemical, thermal and mechanical resistance as well as its processability. Indeed, although standard grades of DGEBA (*n* ≈ 0.2) already display a satisfactory range of viscosity of 11.000–16.000 mPa s at 25 °C, medium (*n* ≈ 0.1) and low viscosity grades (*n* < 0.1) defined respectively by viscosity ranges of 7.000–10.000 and 4.000–6.000 mPa s have been developed to facilitate its use in low temperature processes.^1^ Nevertheless, this decrease in viscosity is predominantly accompanied by an increase in cost, rigidity and a drop in adhesion properties leading to stiffer and more fragile materials. Thus, to compensate for this drawback, diluents are usually used in combination with standard grades of DGEBA.

Typical reactive diluents are low molecular weight compounds with reduced viscosity at room temperature and with at least one reactive function to be incorporated into the resulting thermoset material network. Reactive diluents play a significant role in epoxy resin industry by paving the way towards process or applications requiring low viscosities and improved mechanical performances in terms of flexibility, elongation or impact resistance for instance.^2^ Recent requirements toward low volatile organic compound (VOC) and low carbon impact stressed out their utilization and the development of new series prepared from renewable resources. Common current commercial biobased glycidyl ether reactive diluents include dodecyl and tetradecyl glycidyl ethers (C_12_–C_14_ MGE), cardanol monoglycidyl ether (CMGE) and butanediol diglycidyl ether (BDGE) which can be respectively derived from vegetable oil,^3^ cardanol (cashew nutshell liquid)^4^ and 1,4-butanediol (sugar fermentation).^5^ Although produced at industrial level, these biobased reactive diluents can suffer from limited availability and can lead to thermoset materials with reduced stability and thermomechanical properties due to their aliphatic structures. For these reasons, there is still an important need to develop alternative biobased epoxy reactive diluent with advanced properties to compete with current commercial analogues and open up new possibilities.

In this context, vegetable oils (VOs) are one of the most advanced solutions for the preparation of biobased epoxy reactive diluents due to their abundancy and low-cost as well as their strong diversity.^6^ S. Kumar *et al.*^7^ and F. I. Altuna *et al.*,^8^ separately investigated the influence of epoxidized soybean oil (ESO) onto the thermal and mechanical properties of DGEBA-based materials. In both cases, an anhydride hardener was used along with an imidazole-based catalyst. Overall results showed that increasing the ESO concentration leads to thermoset materials with reduced glass transition temperature, thermal stability and reactivity but effectively result in materials with improved fracture toughness and impact strength up to 38%.^8^ However, the impact of ESO's concentration onto the formulations' viscosity was not discussed. Plus, VOs still exhibit long aliphatic chains which could raise several issues as described before. Mo J. Ding *et al.*^9^ reported the synthesis of furfuryl glycidyl ether (FGE) and investigated the impact of its concentration on the thermomechanical properties of DGEBA-based formulations. It was evaluated that best ratio of FGE is around 10 to 15 wt% to increase the flexural strength and the tensile strength of around 16% without affecting the glass transition temperature (*T*_g_) of the resulting thermoset material. In a similar approach, Z. Karami *et al.*^10^ combined FGE with DGEBA in weight concentration up to 30%. Interestingly, it was put forward that FGE has stronger viscosity-reducing ability than a commercial reactive diluent known under the brand name of Cardura E10P (Momentive). Furthermore, once cured with diethylene triamine (DETA), it was concluded that the use of 20 wt% of FGE results in materials with increased adhesion (up to 300%), tensile (up to 40%) and flexural strengths (up to 60%). In a study from J. Chen *et al.*,^11^ the authors were interested in the synthesis and use of a polyepoxidized cardanol glycidyl ether (PECGE) as new biobased reactive diluent of DGEBA resin. When compared to its CMGE counterpart, the PECGE showed higher viscosity (+27%) but still satisfactory value of 2150 mPa s was obtained when this diluent was used at 20 wt% in combination with DGEBA. Finally, it was concluded that from an application point of view, the formulations containing 10 to 15 wt% of PECGE combines both improved processability and materials thermomechanical properties. Finally, eugenol was also largely considered due to its availability as well as its peculiar structure allowing to yield biobased reactive diluent with various properties.^12^ For instance, A. Maiorana *et al.*^13^ reported the synthesis of the monoglycidyl ether of eugenol (MGEu) which was further combined with the Diglycidyl Ether of Diphenolate Pentyl Ester (DGEDP-Pe) as DGEBA substitute. Interestingly, MGEu showed viscosity of 25 mPa s at 25 °C allowing to drastically reduce the formulation's viscosity from 11.5 Pa s to almost 1 Pa s when 20 wt% was added to the neat resin. However, after curing with isophorone diamine (IPDA), this blend led to a thermoset material with very limited *T*_g_ of 65 °C.

Although some interesting data can be extracted from the literature, the research towards the development and assessment of new classes of epoxy-based reactive diluents remain very scarce. Moreover, it could be shown that some of these compounds exhibit an aliphatic structure with internal aliphatic epoxy functions. If this can negatively affect the properties of the final material from thermal stability and thermomechanical properties viewpoints, it is also important to note that this can lead to a difference in reactivity and thus to a heterogeneity or incomplete epoxy conversion resulting in lower cross-linking density in the final material.

Taking this into consideration, this study was dedicated to the synthesis and evaluation of a new range of biosourced epoxy reactive diluents prepared from natural phenols carvacrol, thymol and guaiacol which were already used as interesting diluting candidates in vinyl ester^14^ and epoxy^15^ resins. Such monophenols can be respectively isolated from oregano and thyme essential oils^16–18^ and lignin^19^ for instance. Biobased thymol and carvacrol can be also produced from largely available natural resources such as limonene and cymene.^20^ Their viscosity and the influence of their loading on DGEBA's viscosity were evaluated. In addition, DSC measurements were conducted on each formulation mixed with IPDA to investigate how the diluent content would affect the reactivity and associated glass transition temperature of the final cured material. Commercial epoxy reactive diluents C_12_–C_14_ MGE, CMGE, BDGE and commercial DGEBA-based epoxy resins prepared therefrom were used as reference to compare the results obtained from this study.^21^

## Results and discussion

### Synthesis and characterization of epoxy diluents

**Fig. 1:**
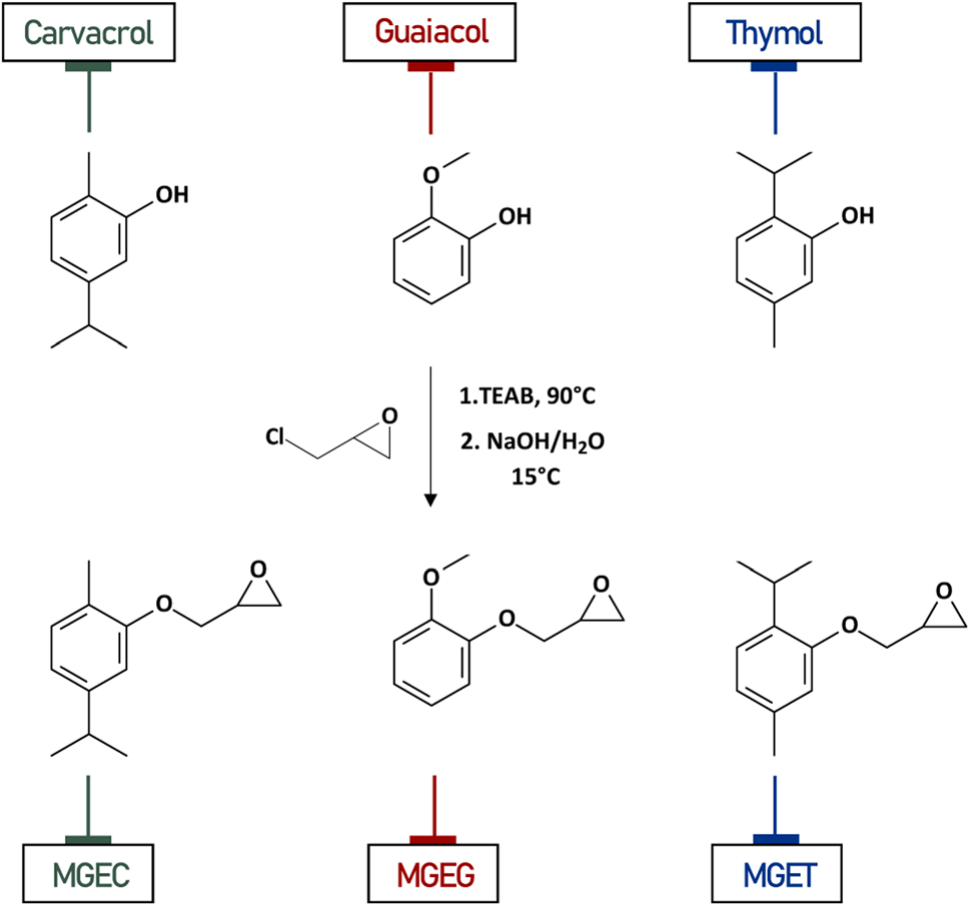
Synthesis pathway of MGEC, MGEG and MGET from carvacrol, guaiacol and thymol respectively.

The synthesis of monofunctional reactive diluents (MGEG, MGEC and MGET) was carried out using an efficient general two steps one-pot procedure (Fig. 1) extensively used in industry and described in detail in the dedicated section.

Phenol derivatives (guaiacol, carvacrol and thymol) were first put in contact at 90 °C with an excess of epichlorohydrin using tetraethylammonium bromide (TEAB) as phase transfer catalyst followed by the addition of a NaOH alkaline solution at 15 °C. According to previous work from S. Caillol's group,^22,23^ two main reactions can co-exist during the first reaction step. In the first case, a S_N_2 reaction is performed by the phenolate ion onto the chlorine-carrying carbon to directly yield the corresponding epoxide compound. The second side-reaction involves a nucleophilic attack of the phenolate ion onto the electrophilic methylene carbon of the epichlorohydrin oxirane, thereby ensuring the opening of the oxirane ring. Here, the 2^nd^ step using alkaline solution ensures the internal closure of the oxirane ring with a retention of configuration.

**Fig. 2:**
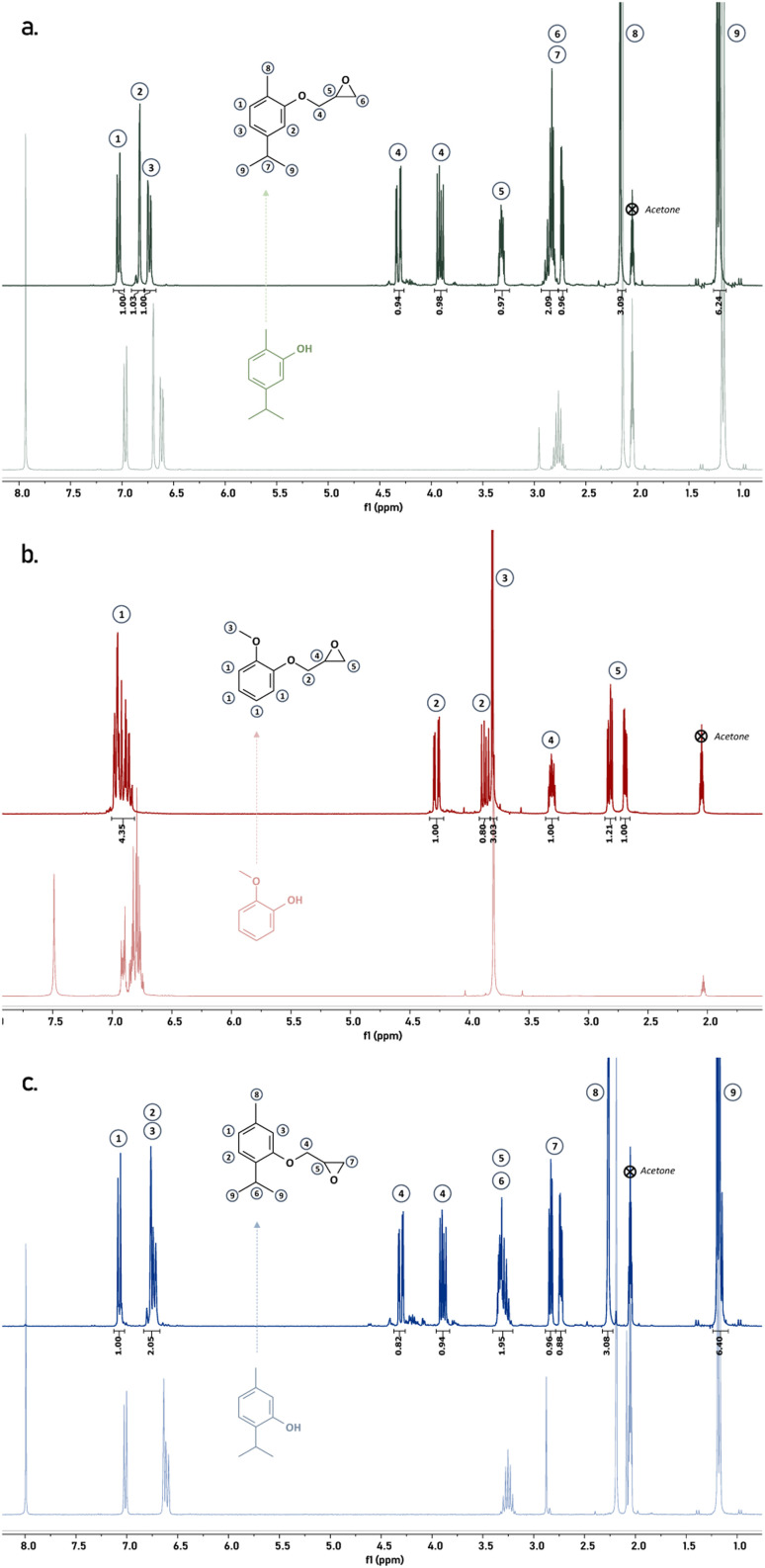
Overlay of ^1^H NMR spectra of carvacrol (light green) and MGEC (dark green) (a), guaiacol (light red) and MGEG (dark red) (b) and thymol (light blue) and MGET (dark blue) (c).

This process allowed us to obtain the epoxides with good to very high yields ranging from 79 to 99%, which is close or even better to results already reported.^9,10,13^ Obtained products were finally characterized by means of ^1^H NMR (Fig. 2) and FT-IR (Fig. S1 – see ESI†). Overlay of ^1^H NMR spectra of the raw materials with the corresponding epoxides highlight the complete conversion of the phenols into epoxides according to the chemical shifts of each peak and the presence of the characteristic peaks of the oxirane groups between 4.5 and 2.5 ppm.

In addition, FT-IR spectra of the feedstocks display a broad signal between 3700 and 3100 cm^−1^ which was attributed to the stretching vibration of the –OH of the phenol derivatives. Its complete disappearance on the FT-IR spectrum of the epoxide products gives confirmation of phenols conversion. Glycidyl ether functions can be here confirmed by the signals at 912 cm^−1^ attributed to the C–O stretching of the oxirane ring as well as the intense signal between 1040 and 1015 cm^−1^ corresponding to the stretching of the ether (C–O–C) linkage.

### Comparison of epoxy reactive diluents' viscosity

As already mentioned, the viscosity of epoxy resins is an important factor that determines its processing conditions. Apart from the use or development of epoxy with low intrinsic viscosity, addition of reactive diluents to conventional epoxies remains one of the more straightforward approaches to adjust this parameter. The viscosity of each reactive diluent as a function of temperature was thus evaluated by a rheometer as given in Fig. S2a (see ESI†). Overall results are gathered in the Table 1. By comparing their viscosity at 20 °C, developed biobased epoxy diluents can be ranked by order of increasing viscosity as followed MGEC ≈ MGET < MGEG with respective viscosity of 16, 17 and 55 mPa s. The lower viscosities obtained for MGEC and MGET compared to MGEG could be explained by the presence of methyl and isopropyl substituents on their structure which would reduce the hydrogen interactions between oxirane functions. This phenomenon has been previously highlighted in a study by M. D. Garisson *et al.*,^24^ where they found that these substituents also reduce water uptake by preventing the formation of polar hydrogen bonding sites. In addition, the study of E. D. Hernandez *et al.*^25^ put forward the ability of the oxygen of the methoxy moiety to participate in hydrogen bonding. The combination of both phenomena would easily explain the rheological behavior of MGEG which exhibits a viscosity at 25 °C more than 3 times higher than MGEC and MGET.

**Table 1:** Viscosity of the reactive diluents at different temperatures

Sample	Viscosity (mPa s)					
	20 °C	25 °C	30 °C	40 °C	50 °C	60 °C
BDGE	10	7.9	6.8	5.3	4.3	3.5
C12–C14 MGE	9.5	7.7	6.7	5.3	4.4	3.7
CMGE	104	81	62	39	26	19
MGEC	16	12	9.8	6.6	4.6	3.5
MGEC-P	12	9.6	7.7	5.3	3.9	3.0
MGEG	55	38	27	15	9.7	7.6
MGET	17	13	10	6.7	4.7	3.4

Above all, it is worth noticing that their viscosities at 25 °C are in the same range of commercial ones such as BDGE (7.9 mPa s), C_12_–C_14_ MGE (7.7 mPa s) and CMGE (81 mPa s) and even lower to other epoxy reactive diluents reported in the literature,^11,13^ providing compelling evidence of their ability to be used as diluents. Also, it is worth mentioning that viscosities of MGEC and MGET start being lower than C_12_–C_14_ MGE and BDGE at higher temperatures *i.e.* in the range of 55–60 °C.

In addition, the influence of the purity of the reactive diluents on their viscosity was evaluated using the MGEC as a reference. Distillation was carried out to ensure the separation of the product from its impurities which were predominantly constituted of oligomeric MGEC fractions. A purified grade of MGEC (MGEC-P) was thus isolated in a respective yield and purity of 77% and 98%. Interestingly, MGEC-P showed reduced viscosity to 12 mPa s at 20 °C (Fig. S2c†) which was found to be even lower than those of commercial BDGE and C_12_–C_14_ MGE as soon as temperatures above 40 °C were reached.

### Effect of diluent concentration on DGEBA's viscosity

**Fig. 3:**
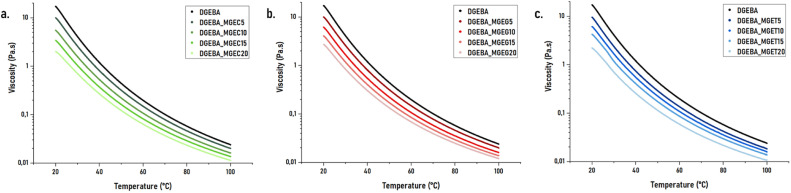
Viscosity as a function of temperature of DGEBA_MGEC (a), DGEBA_MGEG (b) and DGEBA_MGET (c) mixtures.

The reactive diluents developed were found to have interesting low viscosities, which would result in significant diluent abilities when mixed with high viscosity resins. In this regard, each reactive diluent was mixed with DGEBA in weight ratios ranging from 5 to 20 wt% according to Table S1.† The rheograms displayed in Fig. 3a, b and c give respectively the loading effect of MGEC, MGEG and MGET onto DGEBA's viscosity. Unsurprisingly, the latter decreases with the increasing of diluent concentration. As a result, significant drop from approximately 17 000 to 10 000 mPa s can be observed as soon as 5 wt% of reactive diluent were added to DGEBA with a minimal viscosity of around 2000 mPa s reached when DGEBA was diluted with 20 wt% of MGEC.

Obtained results show, in general, similar trends of dilution effect observed on neat reactive diluents. Therefore, except for concentration of 5 wt% in DGEBA (DGEBA_MGEC5), MGEC leads to lower viscosities than its counterparts. By contrast and despite the very similar viscosity of MGET compared to MGEC, DGEBA/MGET mixtures possess viscosity at 20 °C from 10 to 20% higher than those based on MGEC. Finally, although MGEG displayed initial higher viscosity (Fig. S2a† and Table 1), similar or even higher dilution effect than MGET are obtained when MGEG's loading is lower than 15 wt%. Looking at MGEC-P, it was found that its reduced intrinsic viscosity makes it possible to significantly decrease the viscosity of the DGEBA where a drop of up to 25% at 25 °C was observed comparatively to unpurified MGEC-based mixtures.

Comparison of developed mixtures with commercial reference resins (Fig. S2b†) shows that a weight loading of 15 to 20% for each reactive diluent is required to reach similar to lower viscosities at room temperature. By contrast, weight loading of 10 to 15% of MGEC-P (Fig. S3 and Table S1†) can be sufficient to reach comparable viscosity to DGEBA_BDGE and DGEBA_MCGE used as references. DSC measurements were therefore carried out to assess whether the use of the developed products in such concentration levels does not have a detrimental impact on the glass transition temperatures (*T*_g_s) of the final cured materials. Overall data are given and discussed in the following section.

### Reactivity and glass transition temperature of formulated systems

The reactivity of an epoxy is not only governed by the nature and structure of the hardener but also by the epoxy itself. As highlighted in a review from A-S. Mora *et al.*,^26^ electron-withdrawing inductive effect and N- and O-hetero-elements in β position of the epoxy ring allow increasing the reactivity of the epoxy toward nucleophilic attacks. Selecting epoxides with similar reactivities is therefore a fundamental axis to ensure material curing under mild conditions but also to avoid limited epoxy conversion which can lead to materials with reduced stability and thermo-mechanical properties or even premature aging.^4,27^

**Fig. 4:**
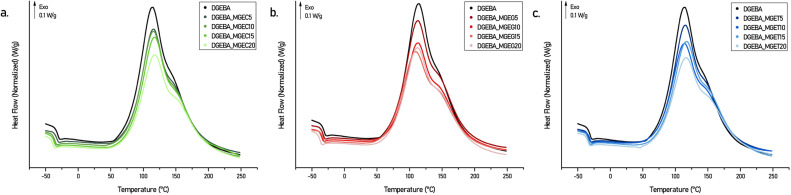
Non-isothermal DSC curves of DGEBA/MGEC (a), DGEBA/MGEG (b) and DGEBA/MGET (c) systems mixed with IPDA.

**Fig. 5:**
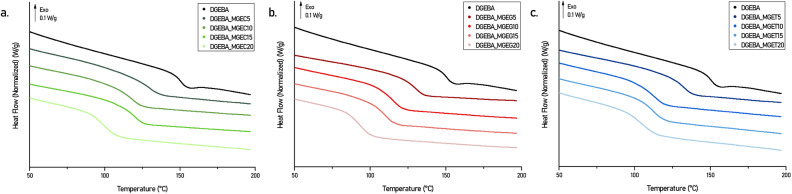
DSC thermograms (second heating cycle) of DGEBA/MGEC (a), DGEBA/MGEG (b) and DGEBA/MGET (c) systems cured with IPDA in an oven.

Non-isothermal curing behaviours of the DGEBA-based systems mixed with IPDA were studied by differential scanning calorimetry (DSC) (Fig. 4, S4a and S5a†) and the main data are collected in Table S2.† Each DSC curve exhibits a major exothermic peak corresponding to the curing reaction with a noticeable shouldering at higher temperature. The latter was attributed to the difference in nature and therefore in reactivity between the aliphatic and cycloaliphatic amines beared by the IPDA.^28,29^ Nevertheless, this phenomenon is observed for each system with similar intensity and proportion which, combined with the fact that the *T*_onset_ and *T*_peak_ obtained are also close to each other regardless the concentration and nature of the reactive diluent, confirm that developed epoxy compounds have similar reactivity. Further DSC studies were carried out on oven cured materials. Fig. 5 shows the DSC thermograms obtained on the 2^nd^ heating ramp of the developed systems. First, it is worth mentioning that no exothermic signal was observed on the 1^st^ DSC heating ramps confirming the adequacy of the curing cycle used to obtain a maximum of crosslinking. Furthermore, the *T*_g_s obtained on the oven-cured materials are in perfect adequacy with those obtained on the 2^nd^ heating ramp carried out on the formulations during the study of their curing behaviours. DGEBA/IPDA reference system shows a *T*_g_ at 149 °C which is in good agreement with previous results reported in the literature.^28,30^ This value starts to decrease by about 20 °C to reach *T*_g_ of 130 °C as soon as 5 wt% of reactive diluent are added. By contrast, maximum of 20 wt% diluent loading leads to minimal but still satisfactory *T*_g_ of 92.7 °C to 104.6 °C for DGEBA_MGEG20/IPDA (Fig. 5b) and DGEBA_MGET20/IPDA (Fig. 5c) systems. More interestingly, while increasing the diluent concentration from 10 to 15 wt%, the *T*_g_ values remain unchanged for DGEBA/MGEC (116.8 *vs.* 118.5 °C) (Fig. 5a) and DGEBA/MGET (110.9 *vs.* 111.7 °C) cured systems or only slightly affected for DGEBA/MGEG (113.2 *vs.* 108.8 °C) material. This could represent an optimal composition to maintain sufficient mechanical stiffness while significantly improving the processability of the resin, which could then compete directly with current commercial reference resins. In that sense, reference systems were mixed with IPDA and cured in an oven following the same curing cycle of 50 °C for 1 h and 130 °C for 1 h 30. As a result and according to Fig. S4b and Table S2,† maximum *T*_g_s of around 126 °C are obtained for the DGEBA_BDGE and DGEBA_C_12_–C_14_ MGE based networks while the DGEBA_MCGE system leads to the lowest *T*_g_ of 102 °C. This latter is close to the *T*_g_s obtained with DGEBA_MGEC20 (*T*_g_ = 99.9 °C) and DGEBA_MGET20 (*T*_g_ = 104.6 °C) systems, which have lower viscosities at 25 °C of 30% and 13% respectively. By substituting the DGEBA_MCGE by the DGEBA_MGEC15 which has similar viscosity at 25 °C (1737 *vs.* 1892 mPa s), an increase in *T*_g_ of 16 °C is obtained. On the other hand, the *T*_g_s of the reference materials DGEBA_BDGE and DGEBA_C_12_–C_14_ MGE are obtained for systems composed of MGEC, MGEG and MGET in proportion barely exceeding 5% by weight. At this concentration level, developed formulations would exceed by 3 to 4.5 times their viscosity at 25 °C.

Finally, it is interesting to highlight that the use of purified epoxy reactive diluents could address the differences in performance observed. Indeed, in the case of DGEBA/MGEC-P systems, resulting thermoset materials cured with IPDA showed *T*_g_s still above 111 °C when 20 wt% of MGEC-P were used (Fig. S5b†). More importantly, comparable viscosity at room temperature and *T*_g_ of DGEBA_BDGE system can be obtained thanks to the use of DGEBA_MGEC-P15 mixture which led to associated values of 1507 mPa s at 25 °C and 124.4 °C respectively.

## Conclusions

A new series of 3 monofunctional epoxies derived from natural phenols namely carvacrol, guaiacol and thymol was developed and assessed as reactive diluents. Their conversion into epoxies was ensured by a general glycidylation procedure allowing to obtain the corresponding products with high yield (>79%) and purity (≈90%) without the use of advanced purification. Through rheology measurements, it has been revealed that these innovative reactive diluents have viscosity values between 12 and 38 cPs at 25 °C, falling within the viscosity range (7.7 to 81 cPs) of the commercial biobased diluents identified in this study. Additional purification of MGEC by distillation (MGEC-P) made it possible to further reduce its viscosity to 9.6 mPa s at room temperature.

Their diluting effect was also evaluated when combined in different weight ratios ranging from 5% to 20% with DGEBA. By selecting the diluent of interest and finely adjusting its proportion, it has been proven that it is possible to drastically reduce the initial DGEBA's viscosity by a factor of almost 10 with viscosities at 20 °C of approximately 2000 cPs when diluent mass ratios reach 20%. Beyond this drop of viscosity highlighting their ability to be used as a reactive diluents, the resulting materials from these formulations crosslinked with IPDA showed glass transition temperatures above 90 °C. Again, the advantages of using the purified grade MGEC-P was highlighted with, first, a reduction of up to 25% of the viscosity at 25 °C of resulting formulations compared to the ones prepared from the unpurified grade MGEC and, finally, an increase of *T*_g_ values of 5 to 15% obtained on cured materials.

Overall results pave the way towards the development of biosourced epoxy formulations with adjustable rheological and thermal properties to meet industrials' requirements.

## Experimental section

### Materials

Thymol (>98%) and tetraethylammonium bromide (TEAB, 98%) were purchased from Alfa Aesar. Carvacrol (natural, 99%, FG), guaiacol (natural, >99%, FG), epichlorohydrin (>99%), anhydrous sodium sulfate (Na_2_SO_4_, 99%), sodium hydroxide pellets (NaOH), isophorone diamine (IPDA) (>99%, AHEW = 42.6 g eq^−1^), 1,4-butanediol diglycidyl ether (BDGE) (>95%), dodecyl and tetradecyl glycidyl ethers (C_12_–C_14_ MGE) (technical grade) and diethyl ether (>95%) were purchased from Sigma-Aldrich. All reagents, reactants and solvents were used as received without further purification. Commercial epoxy resins SR GREENPOXY 28 (SR G28) and SR GREENPOXY 33 (SR G33) were purchased from Sicomin. Formulite 2500 A (F 2500A) and LITE 2513HP (cardanol monoglycidyl ether (CMGE)) were them supplied by Cardolite. Araldite LY 1568 (ALY 1568) was provided by Samaro. Once received, all formulations were thoroughly characterized by means of ^1^H NMR and ^1^H qNMR to evaluate their composition and epoxy content. The following table (Table 2) gives an overview of their main characteristics.

**Table 2:** Main characteristics of selected commercial reference DGEBA-based epoxy resins

Resin	Component 1		Component 2		EI	EEW
	Type	mol%	Type	mol%	meq g^−1^	g eq^−1^
SR G28^a^	DGEBA	100			5.59	178.9
SR G33^b^	DGEBA	80	BDGE	20	5.55	180.2
F 2500A^c^	DGEBA	82	CMGE	18	4.81	207.9
ALY 1568	DGEBA	85	C_12_–C_14_ MGE	15	5.34	187.3

a Produced with 28% of carbon originating from biomass.

b Produced with 35% of carbon originating from biomass.

c Produced with 20% of carbon originating from biomass.

### Epoxidation of phenolic-based derivatives

#### Monoglycidyl ether of guaiacol (MGEG)

The synthesis parameters and applied conditions were used according to previous internal experiences.

Typically, a 1 L three-necked round bottom flask equipped with a thermometer, a reflux condenser and a magnetic stirrer was charged with guaiacol (100 g, 0.81 mol, 1.0 eq), tetraethylammonium bromide (TEAB, 6.773 g, 0.032 mol, 0.04 eq) and epichlorohydrin (372.6 g, 4.03 mol, 5.0 eq). The mixture was kept reacting for 9 h at 90 °C. Once completed, the reaction media was cooled down to 15 °C in an ice bath and a solution of NaOH 33 wt% was added dropwise. After 1 h the reaction was further conducted overnight at room temperature. Once completion of reaction, 300 mL of diethyl ether was added. The organic layer was washed three times with water (3 × 150 mL), dried over anhydrous Na_2_SO_4_ and then filtered. Solvents were removed under reduced pressure (10^−1^ mbar) at 60 °C using a rotary evaporator. Monoglycidyl ether of guaiacol (MGEG) was obtained as a clear yellow liquid with a yield of 97% (141.3 g) and a purity of 90%. The epoxy index was evaluated at 4.98 meq g^−1^. A sample was submitted to ^1^H NMR and ^1^H qNMR.

^1^H NMR (300 MHz, acetone-d6) *δ*: 6.94 (m, 4H), 4.29 (dd, 1H), 3.87 (dd, 1H), 3.82 (s, 3H), 3.32 (m, 1H), 2.84 (dd, 1H), 2.71 (dd, 1H).

#### Monoglycidyl ether of carvacrol (MGEC)

MGEC was synthetized following a same procedure to MGEG but starting with carvacrol as phenolic derivative. MGEC was obtained in a 95% yield as a light-yellow liquid with an epoxy index evaluated by ^1^H qNMR of 4.34 meq g^−1^ and a purity of 89%.

A purified grade of MGEC (MGEC-P) was obtained using a distillation setup. The distillation temperature was initially set at 100 °C and gradually increased to 170 °C without vacuum pressure control. MGEC-P was continuously collected between 110 °C and 150 °C. The impurities were evaluated by ^1^H NMR as residual epichlorohydrin (first distilled fraction) and MGEC oligomers (non-volatile fraction).

MGEC-P was isolated in a 77% yield as a colorless liquid. Its epoxy index and its associated purity were respectively evaluated by ^1^H qNMR at 4.76 meq g^−1^ and 98%.

^1^H NMR (300 MHz, acetone-d6) *δ*: 7.03 (d, 1H), 6.83 (s, 1H), 6.74 (d, 1H), 4.32 (dd, 1H), 3.91 (dd, 1H), 3.32 (m, 1H), 2.84 (q, 1H), 2.84 (dd, 1H), 2.73 (dd, 1H), 2.17 (s, 3H), 1.21 (d, 6H).

#### Monoglycidyl ether of thymol (MGET)

MGET was synthetized following a same procedure to MGEG but starting with thymol as phenolic derivative. MGET was obtained in a 79% yield as a light-yellow liquid with an epoxy index evaluated by ^1^H qNMR of 4.3 meq g^−1^ and a purity of 89%.

^1^H NMR (300 MHz, acetone-d6) *δ*: 7.09 (d, 1H), 6.78 (s, 1H), 6.74 (d, 1H), 4.32 (dd, 1H), 3.91 (dd, 1H), 3.33 (m, 1H), 3.31 (q, 1H), 2.85 (dd, 1H), 2.74 (dd, 1H), 2.28 (s, 3H), 1.21 (d, 6H).

### Preparation of the epoxy mixtures

Reactive diluents (MGEG, MGET, MGEC and MGEC-P) were added at different weight ratios of 0, 5, 10, 15, and 20% to DGEBA (SR GREENPOXY 28) previously weighed in a speed-mixer container (6 mL). Thorough mixing was ensured in a speed mixer using the following program of 3500 rpm for 1 min and 2500 rpm for 1 min. EEW of the mixtures (EEW of mix) was determined using eqn (1):1
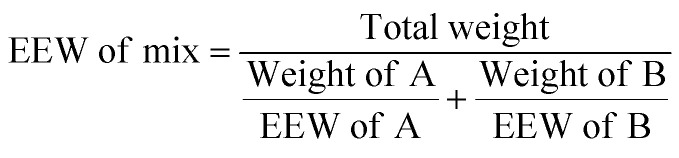


### Preparation of cured epoxy networks

Prepared epoxy mixtures and commercial DGEBA-based resins were formulated with IPDA in stoichiometric ratio calculated from eqn (2) and (3):2
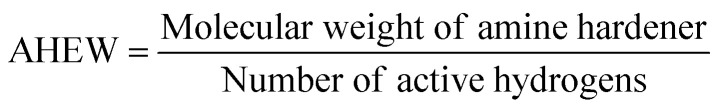
3
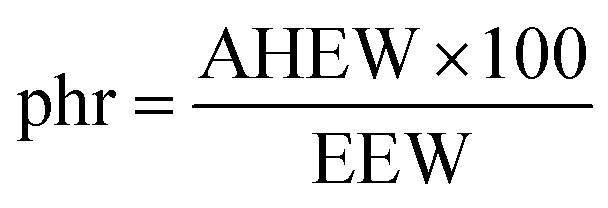
where AHEW (g eq^−1^) is the amine hydrogen equivalent weight and phr (per hundred resin) is the quantity of amine hardener required to cure 100 g of epoxy resin. According to (2), the AHEW of the IPDA was evaluated at 42.6 g eq^−1^.

Each formulation was then carefully mixed in a speed-mixer using a similar program of 3500 rpm for 1 min and 2500 rpm for 1 min. The formulations were then poured in aluminium plates and cured in an oven at 50 °C for 1 h and 130 °C for 1 h 30.

### Methods

#### Nuclear magnetic resonance (NMR)

^1^H NMR spectrum of the synthesized reactive diluents were obtained using Bruker Advance 300 (300 MHz) spectrometer equipped with a QNP probe at room temperature. Acetone-d_6_ was used as deuterated solvent. Chemical shifts (*δ*) are given in ppm referenced to the residual deuterated solvent peak.

The Epoxide Index (EI, meq g^−1^) was determined according to ^1^H NMR titration method (^1^H qNMR). The method consists in solubilizing a known mass of the product and of an internal standard (trioxane with a purity of 99.9%). The number of moles of epoxide per gram of product was measured by comparing the standardized integration of the standard with the standardized integration of the oxirane rings.

The epoxy equivalent weight (EEW, g eq^−1^) was determined thanks to the EI (meq g^−1^) using the following eqn (4):4
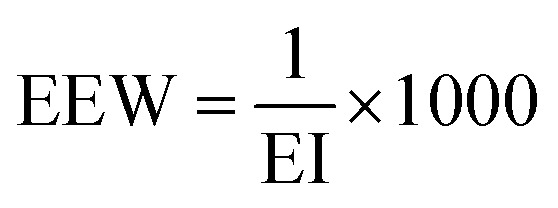


#### Fourier transform infrared (FT-IR)

Fourier Transform Infrared Spectroscopy (FT-IR) of the sample was operated on a spectrum two infrared spectrophotometer (PerkinElmer) with a LiTaO_3_ (lithium tantalate) detector using a Diamond UATR accessory unit.

The sample was then placed directly on the ATR crystal. The spectra were acquired with 16 scans from 4000 to 500 cm^−1^ in transmission mode in air through a resolution of 4 cm^−1^. The Spectrum 10 software from PerkinElmer was used to record and process the data.

#### Differential scanning calorimetry (DSC)

Differential Scanning Calorimetry (DSC) measurements were carried out using DSC Q2000 instrument (TA INSTRUMENTS). The data were recorded with Advantage software and analysed with TRIOS software. The calorimeter was calibrated using an indium standard (heat flow and temperature calibration).

The sample (around 5–7 mg) was placed into 40 μL Tzero hermetic pan with manual pierced lid. Nitrogen at 50 mL min^−1^ was used as a purge gas. Curing behaviour of the formulations was investigated at a heating rate of 10 °C min^−1^ using temperature ramps of (1) heating from −60 to 250 °C; (2) cooling from 250 to 30 °C; (3) heating from 30 to 200 °C. Cured samples were them evaluated at a heating rate of 10 °C min^−1^ using the following temperature cycles: (1) heating from 30 to 250 °C; (2) cooling from 250 to 30 °C; (3) heating from 30 to 200 °C.

#### Rheometry

Rheological properties were measured using an HR20 Rheometer (TA INSTRUMENTS) equipped with a Peltier plate for temperature control. The data were recorded and analyzed with TRIOS software.

The Newtonian domains were evaluated on each sample at minimum and maximum temperature on flow sweep mode with shear rate oscillating between 0.1 and 100 s^−1^. The shear rate was finally set at 10 s^−1^ for all samples. Approximately 1–2 mL of sample was placed on the Peltier plate and the parallel-plate geometry (40 mm) was used in all experiments while the gap was set to 500 μm. Sample trimming was made on samples exceeding parallel-plate size. The viscosity of each sample was measured using a temperature ramp rate of 2 °C min^−1^ from 20 to 60 °C for the reactive diluents and 20 to 100 °C for the formulations.

## Conflicts of interest

There are no conflicts to declare.

## Supplementary Material

RA-013-D3RA01039B-s001
