# Supplementary material for: Biobased epoxy reactive diluents prepared from monophenol derivatives: effect on viscosity and glass transition temperature of epoxy resins
Source: RSC Adv. 2023 May 16;13(22):15099–106. doi: 10.1039/d3ra01039b (PMC10189243; doi:10.1039/d3ra01039b)
Supplement: RA-013-D3RA01039B-s001 [file RA-013-D3RA01039B-s001.pdf]

Electronic Supplementary Material (ESI) for RSC Advances.
This journal is © The Royal Society of Chemistry 2023

# New series of biobased epoxy reactive diluents: effect on viscosity and glass transition temperature of epoxy resins

## Supporting information

**1. FTIR analysis of the phenols and their associated epoxy derivatives**

a.

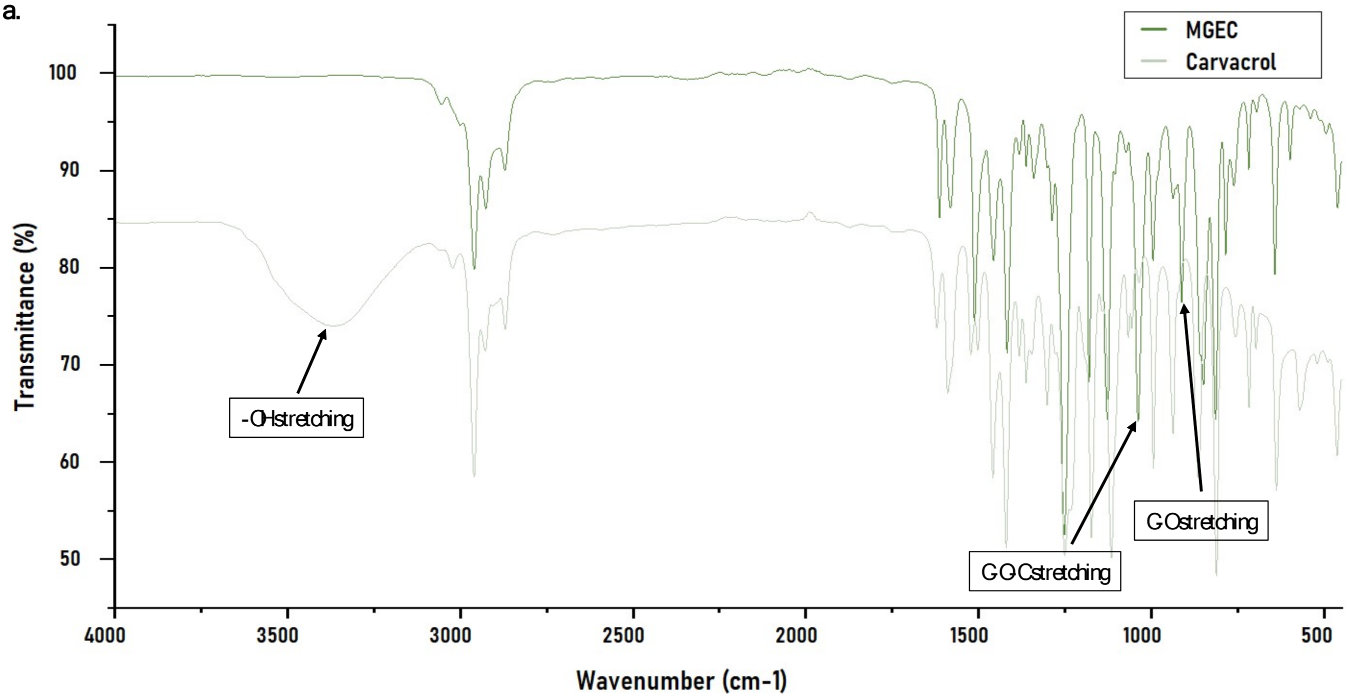


b.

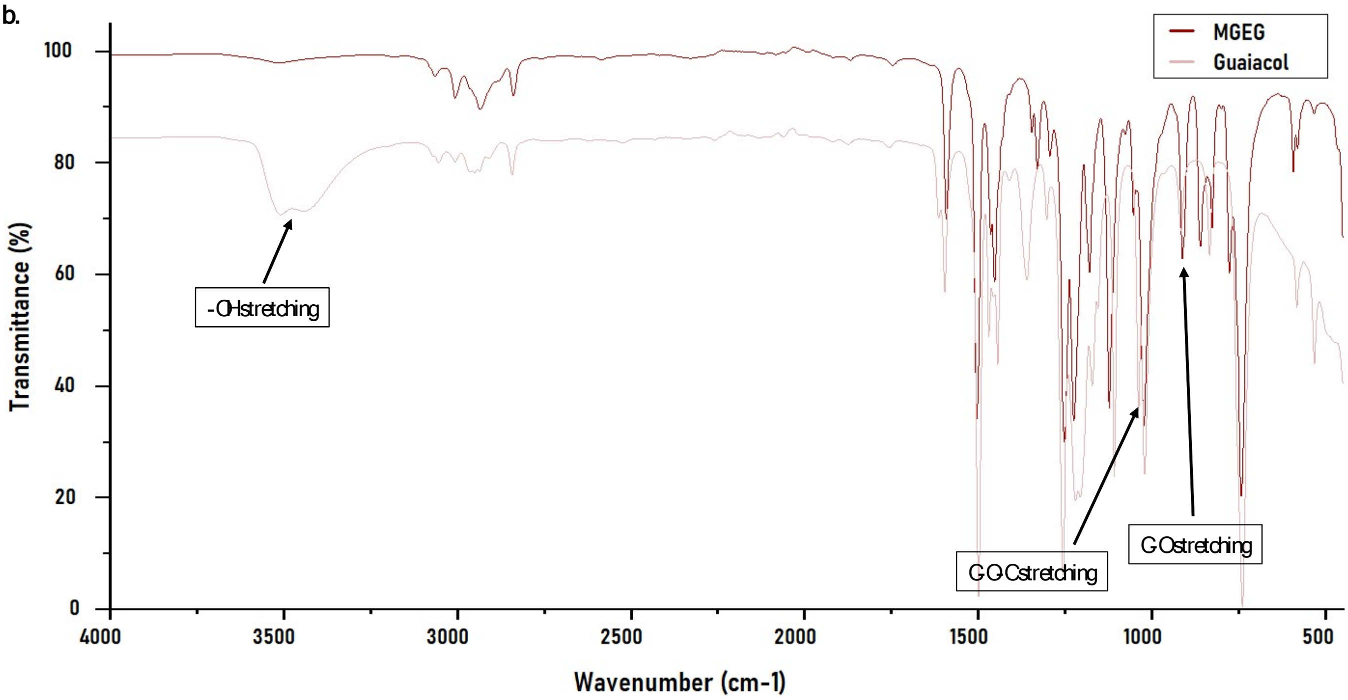

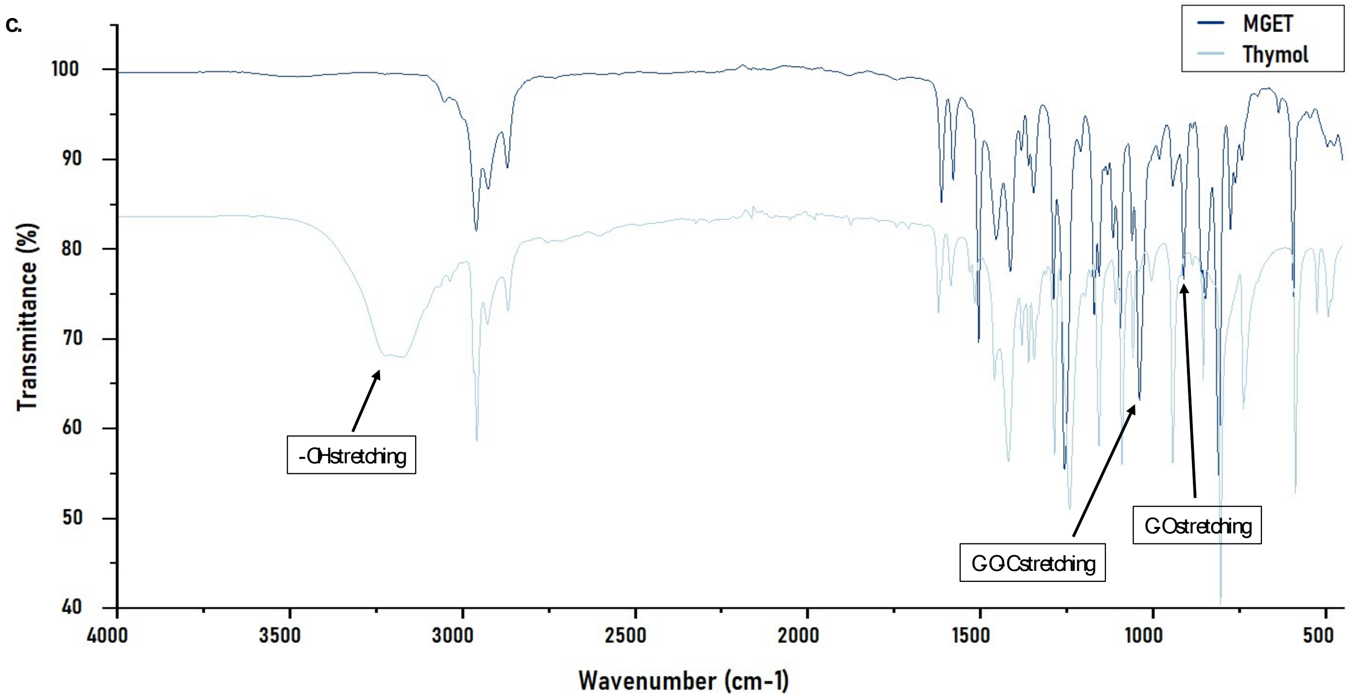


Figure S1. Overlay of FTIR spectra of carvacrol (light green) and MGEC (dark green) (a), guaiacol (light red) and MGEG (dark red) (b) and thymol (light blue) and MGET (dark blue) (c)

## 2. Rheological curves and data

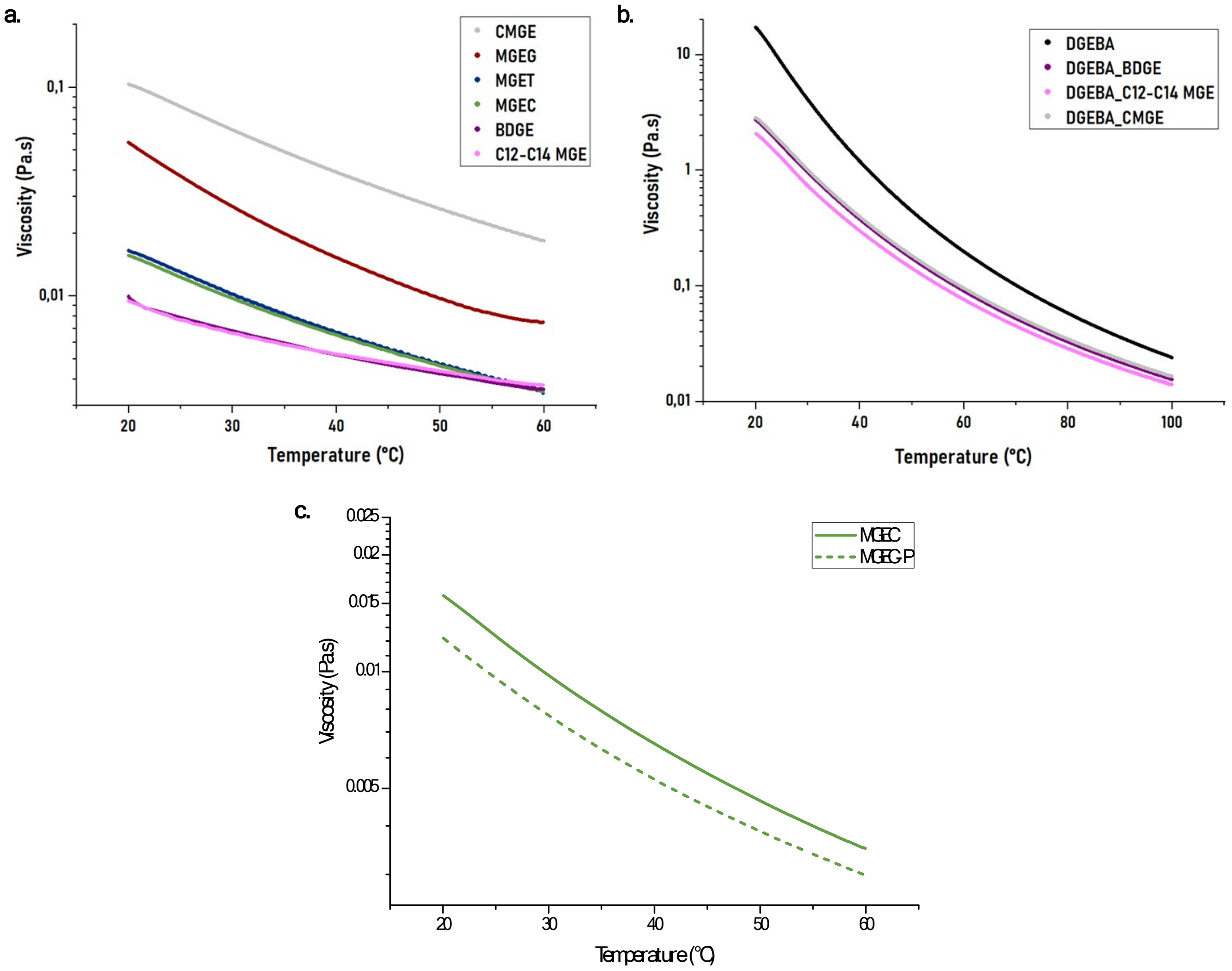


Figure S2. *Viscosity as a function of temperature of each reactive diluent (a), DGEBA-based commercial reference systems (b) and purified MGEC-P (c)*

Table S1. Viscosity of the formulations at different temperatures

| Sample | Viscosity (mPa.s) | | | | | |
|---|---|---|---|---|---|---|
| | 20 °C | 25 °C | 40 °C | 60 °C | 80 °C | 100 °C |
| DGEBA (SR GREENPOXY 28) | 17238 | 8436 | 1195 | 198 | 58 | 24 |
| DGEBA_BDGE (SR GREENPOXY 33) | 2729 | 1639 | 377 | 90 | 33 | 16 |
| DGEBA_MCGE (FORMULITE 2500 A) | 2850 | 1737 | 397 | 96 | 35 | 17 |
| DGEBA_C12-C14 MGE (Araldite LY 1568) | 2074 | 1274 | 299 | 76 | 29 | 14 |
| DGEBA_MGEC5 | 10064 | 5120 | 814 | 149 | 46 | 20 |
| DGEBA_MGEC-P5 | 8519 | 4036 | 627 | 116 | 37 | 17 |
| DGEBA_MGEC10 | 5528 | 2972 | 532 | 107 | 36 | 16 |
| DGEBA_MGEC-P10 | 5656 | 2835 | 494 | 99 | 33 | 15 |
| DGEBA_MGEC15 | 3431 | 1892 | 373 | 82 | 29 | 14 |
| DGEBA_MGEC-P15 | 2861 | 1507 | 295 | 66 | 24 | 11 |
| DGEBA_MGEC20 | 2024 | 1222 | 270 | 64 | 24 | 11 |
| DGEBA_MGEC-P20 | 1605 | 919 | 198 | 48 | 18 | 8.3 |
| DGEBA_MGEG5 | 10051 | 4736 | 807 | 148 | 46 | 20 |
| DGEBA_MGEG10 | 6219 | 3238 | 548 | 107 | 36 | 16 |
| DGEBA_MGEG15 | 4088 | 2364 | 404 | 85 | 29 | 14 |
| DGEBA_MGEG20 | 2744 | 1307 | 304 | 69 | 25 | 12 |
| DGEBA_MGET5 | 9585 | 5110 | 739 | 136 | 42 | 18 |
| DGEBA_MGET10 | 6134 | 3226 | 550 | 109 | 36 | 16 |
| DGEBA_MGET15 | 4261 | 2190 | 407 | 86 | 30 | 14 |
| DGEBA_MGET20 | 2221 | 1519 | 256 | 59 | 22 | 11 |

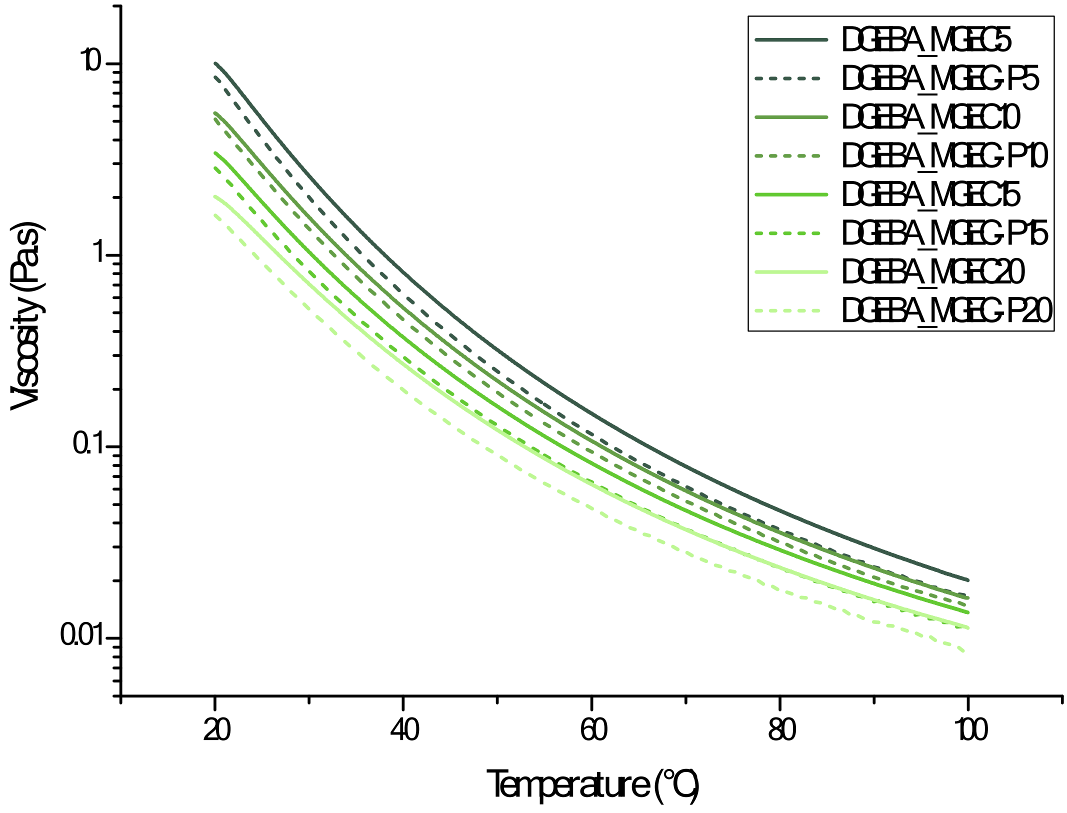


Figure S3. Viscosity as a function of temperature of DGEBA_MGEC and DGEBA_MGEC-P mixtures

## 3. Non-isothermal DSC analysis of epoxy formulations and oven-cured materials with IPDA

Table S2. Main data obtained from the DSC measurements carried out on the formulations and oven-cured materials

| Formulation | EEW | phr | Tonset | Tpeak | Enthalpy | Tg |
|---|---|---|---|---|---|---|
| | g/eq | g | °C | °C | J.g-1 | °C |
| DGEBA (SR GREENPOXY 28) | 178.9 | 23.8 | 84.5 | 117.5 | 407.5 | 149.2 |
| DGEBA_BDGE (SR GREENPOXY 33) | 180.2 | 23.6 | 78 | 115.2 | 457.4 | 127.8 |
| DGEBA_MCGE (FORMULITE 2500 A) | 207.9 | 20.5 | 77.6 | 119.1 | 375.2 | 102.3 |
| DGEBA_C12-C14 MGE (Araldite LY 1568) | 187.3 | 22.7 | 81.8 | 120 | 416.4 | 125.5 |
| DGEBA_MGEC5/IPDA | 180.8 | 23.5 | 79.2 | 116.4 | 396.9 | 128.8 |
| DGEBA_MGEC-P5/IPDA | 180.2 | 23.6 | 85 | 118 | 443 | 144 |
| DGEBA_MGEC10/IPDA | 182.8 | 23.2 | 77.6 | 116.5 | 408.9 | 116.8 |
| DGEBA_MGEC-P10/IPDA | 181.6 | 23.5 | 86 | 120 | 461 | 135.2 |
| DGEBA_MGEC15/IPDA | 185.2 | 23 | 79.2 | 118.1 | 394.2 | 118.5 |
| DGEBA_MGEC-P15/IPDA | 183 | 23.3 | 82 | 119 | 426 | 124.4 |
| DGEBA_MGEC20/IPDA | 187.3 | 22.7 | 78.1 | 118.4 | 360.2 | 99.9 |
| DGEBA_MGEC-P20/IPDA | 184.4 | 23.1 | 84 | 121 | 426 | 111.8 |
| DGEBA_MGEG5/IPDA | 179.9 | 23.7 | 74.6 | 113.5 | 425.4 | 128.3 |
| DGEBA_MGEG10/IPDA | 180.8 | 23.5 | 73.8 | 113.8 | 369.3 | 113.2 |
| DGEBA_MGEG15/IPDA | 181.8 | 23.4 | 68.6 | 110.4 | 363.4 | 108.8 |
| DGEBA_MGEG20/IPDA | 182.8 | 23.3 | 72.3 | 113.1 | 376.6 | 92.7 |
| DGEBA_MGET5/IPDA | 180.8 | 23.5 | 78.4 | 116.2 | 409 | 131.1 |
| DGEBA_MGET10/IPDA | 183.2 | 23.2 | 75.2 | 114.8 | 367.5 | 110.9 |
| DGEBA_MGET15/IPDA | 185.2 | 23 | 77.5 | 118.2 | 401.7 | 111.7 |
| DGEBA_MGET20/IPDA | 187.6 | 22.7 | 75.2 | 117.6 | 354.6 | 104.6 |

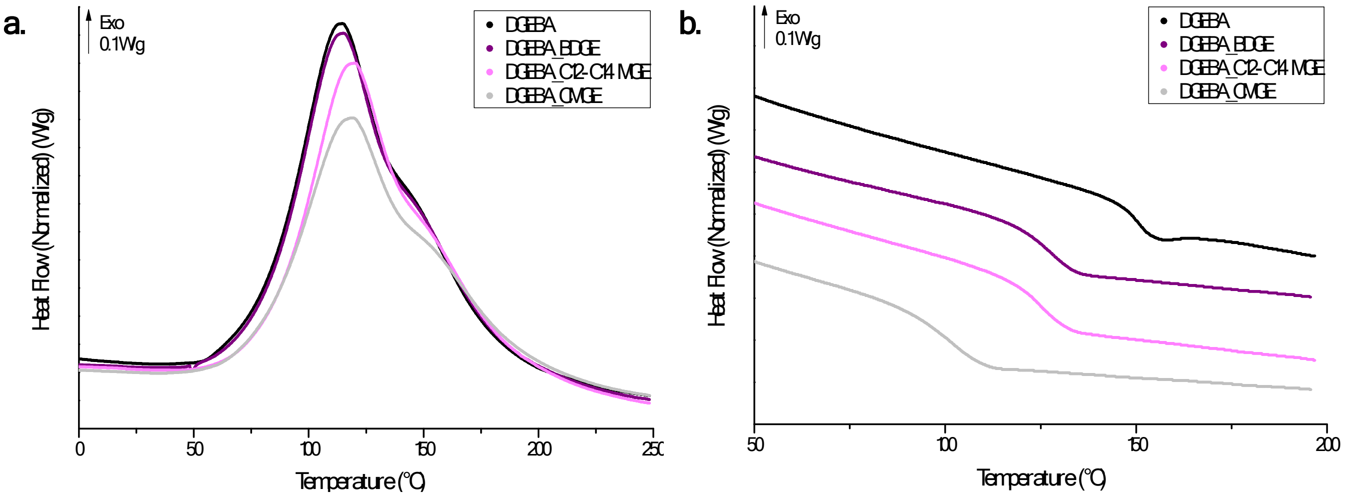


Figure S4. *DSC analysis of uncured (a) and oven-cured (second heating ramp) (b) DGEBA-based commercial reference systems mixed with IPDA.*

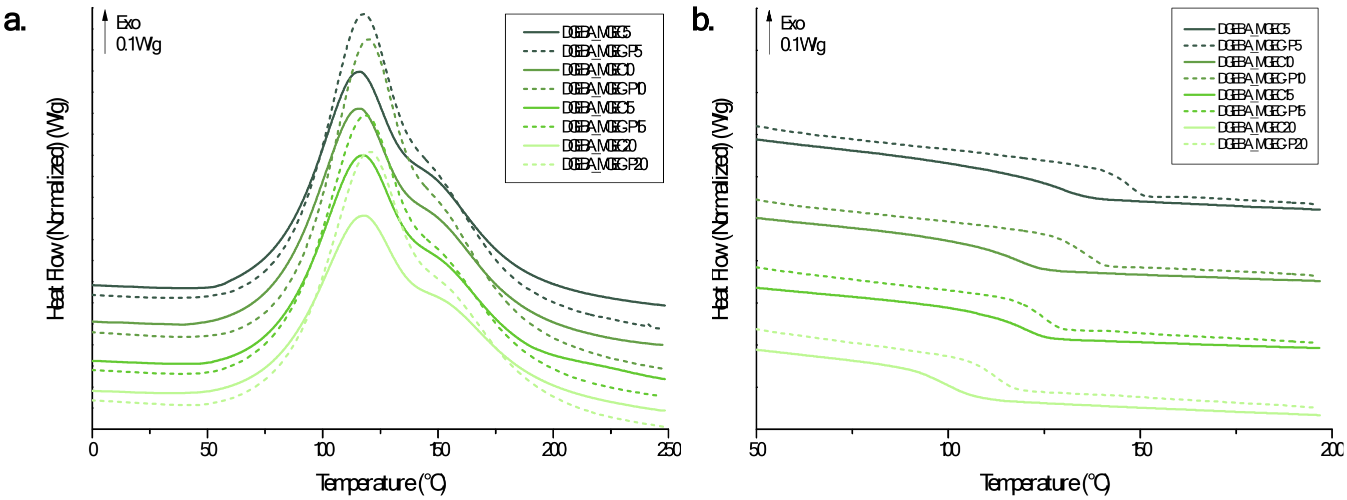

Figure S5. DSC analysis of uncured (a) and oven-cured (second heating ramp) (b) DGEBA_MGEC and DGEBA_MGEC-P systems mixed with IPDA
